# Memorial of the New York City Academy of Medicine

**Published:** 1852-02

**Authors:** 


*Memorial of the New York City Academy of Medicine.*—We have received a memorial by the N. Y. Academy of Medicine to the Legislature of this state, asking for a general law providing for the organization of Medical schools by the managers of public hospitals, or infirmaries having an average number of a hundred patients, or more, with power to grant diplomas. We omit the details, as they will not possess much interest for readers not residing in this state, and most of the practitioners in New York will, it is presumed, be furnished with copies. The object of the memorial purports to be to supply a want of *practical* instruction which can only be obtained at hospitals. The inquiry naturally arises, why not take pains to secure the benefits of hospital instruction in behalf of medical schools already organized? Have they, who have originated, or who favor this new plan, been hitherto conspicuous in their exertions to extend to medical pupils the advantages of clinical teaching? This latter question, as it seems to us, is one which may be very properly and pertinently put. Another inquiry arises, viz., Are the originators and advocates of this new scheme among those who have hitherto been opposed to the multiplication of medical institutions? To require, by a general law, every public hospital, with an average of a hundred patients, to establish medical schools at the request of the State Medical Society, or County Medical Societies, is, at once, to provide for an increase of manufactories of medical practitioners *ad infinitum.* We shall content ourselves, at this time, with merely propounding the foregoing inquiries, without wishing it to be inferred that we are opposed to new medical schools if the profession desire them, and certainly without meaning to imply any hostility to clinical instruction. We fancy that we shall not readily incur suspicion of the latter. On the tendency of the present ferment on the subject of Medical Education, we design to write of some future time. The result appears to us clear. There is a disposition to teach which will not be satisfied while the demand falls so much below the supply. For one we go for the largest liberty in this, as in other matters. We should be glad if all, who are persuaded that their mission lies in this direction, might have ample opportunity to display their capabilities.

In soliciting Legislative action to supply "a want of practical instruction," it strikes us that there is an object which claims precedence of hospital teaching. We allude to legal provisions for anatomical knowledge. If the New York City Academy of Medicine is really desirous of moving in behalf of Medical Education, why not solicit the union of the profession of the State in an application for the legalization of dissections, which, all must admit, form the basis of practical instruction in Medical Science?

